# A Comparison of Primary Human Hepatocytes and Hepatoma Cell Lines to Model the Effects of Fatty Acids, Fructose and Glucose on Liver Cell Lipid Accumulation

**DOI:** 10.3390/nu15010040

**Published:** 2022-12-21

**Authors:** Zoë J. Huggett, Alison Smith, Nicola De Vivo, Dhanny Gomez, Preeti Jethwa, John M. Brameld, Andrew Bennett, Andrew M. Salter

**Affiliations:** 1 Division of Food, Nutrition and Dietetics, School of Biosciences, Sutton Bonington Campus, University of Nottingham, Loughborough LE12 5RD, Leicestershire, UK; 2 Fund for the Replacement of Animals in Medical Experiments (FRAME) Laboratory, School of Life Sciences, University of Nottingham, Nottingham NG7 2UH, Nottinghamshire, UK; 3 National Institute for Health and Care Research (NIHR), Nottingham Biomedical Research Centre, Nottingham NG7 2UH, Nottinghamshire, UK; 4 Future Food Beacon, Sutton Bonington Campus, University of Nottingham, Loughborough LE12 5RD, Leicestershire, UK

**Keywords:** fatty acids, fructose, glucose, hepatocyte, hepatoma cells, non-alcoholic fatty liver disease

## Abstract

Non-alcoholic fatty liver disease (NAFLD) begins with lipid accumulation within hepatocytes, but the relative contributions of different macronutrients is still unclear. We investigated the impact of fatty acids, glucose and fructose on lipid accumulation in primary human hepatocytes (PHH) and three different cell lines: HepG2 (human hepatoblastoma–derived cell line), Huh7 (human hepatocellular carcinoma cell line) and McA-RH7777 (McA, rat hepatocellular carcinoma cell line). Cells were treated for 48 h with fatty acids (0 or 200 μM), glucose (5 mM or 11 mM) and fructose (0 mM, 2 mM or 8 mM). Lipid accumulation was measured via Nile Red staining. All cell types accumulated lipid in response to fatty acids (*p* < 0.001). PHH and McA, but not HepG2 or Huh7 cells, accumulated more lipid with 11 mM glucose plus fatty acids (*p* = 0.004, fatty acid × glucose interaction, for both), but only PHH increased lipid accumulation in response to fructose (*p* < 0.001). Considerable variation was observed between PHH cells from different individuals. Lipid accumulation in PHH was increased by insulin (*p* = 0.003) with inter-individual variability. Similarly, insulin increased lipid accumulation in both HepG2 and McA cells, with a bigger response in McA in the presence of fatty acids (*p* < 0.001 for fatty acid × insulin). McA were more insulin sensitive than either HepG2 or Huh7 cells in terms of AKT phosphorylation (*p* < 0.001 insulin × cell type interaction). Hence, glucose and fructose can contribute to the accumulation of lipid in PHH with considerable inter-individual variation, but hepatoma cell lines are not good models of PHH.

## 1. Introduction

Non-alcoholic fatty liver disease (NAFLD) is estimated to affect 25% of the world’s adult population [1]. It is defined as the presence of hepatic steatosis (lipid accumulation) in greater than 5% of hepatocytes, but in the absence of secondary causes, such as excessive alcohol consumption (>30 g/day for men or >20 g/day for women) [2,3]. NAFLD is frequently associated with obesity, type 2 diabetes and insulin resistance [4]. However, diet is also believed to play a significant role in its development, with diets rich in total energy, high saturated fatty acid and simple sugars all reported to increase hepatic steatosis [5]. Of the dietary sugars, fructose intake has received particular attention, and dietary studies in both animal models [6] and humans [7,8,9,10,11,12] suggest it may have more impact than glucose [7]. Several mechanisms have been proposed to explain the differential effects of dietary fructose and glucose, but further evidence is required to better understand whether this is through direct effects on hepatic lipogenesis [13].

Establishing an appropriate cellular model in which to directly determine the impact of nutrients on hepatic lipid accumulation is challenging. Due to the inherent risk of performing biopsies, it is difficult to access human liver for isolation of ex vivo primary hepatocytes. Liver biopsy is normally only performed on patients already suffering from liver disease (usually cancer), and pharmacological and nutritional treatment prior to surgery may impact on cell viability and functionality. Furthermore, genotypic and phenotypic differences between individuals may impact on the existing level of steatosis within the isolated cells. While primary animal hepatocytes have frequently been used to study hepatic lipid metabolism, the translatability of such findings to humans can be questioned, as major differences in lipid and lipoprotein metabolism between species have been reported [14]. Human hepatoma cell lines, particularly HepG2 and Huh7 cells, have frequently been used as models of hepatic lipid accumulation [15,16,17]. While they offer the advantages of easy cryopreservation and continuous proliferation, the energy requirements of such proliferation may result in major differences in metabolism compared to terminally differentiated primary hepatocytes (which no longer proliferate). The reliance of hepatoma cell lines upon anaerobic glycolysis and subsequent production of lactate, rather than oxidative phosphorylation or fatty acid beta oxidation, may also result in different responses to carbohydrate energy sources compared to primary hepatocytes. Another major disadvantage is their low capacity to secrete very low density lipoproteins (VLDL) [18]. McA-RH7777 (McA) cells are a rat-derived hepatoma cell line and have the potential benefit of being able to secrete VLDL particles [19], but face the same fundamental differences associated with primary animal hepatocytes.

In the present study, we investigated the impact of different concentrations of fatty acids, glucose and fructose on the accumulation of lipid in primary human hepatocytes (PHH), HepG2, Huh7 and McA cells. We also explore the role of insulin sensitivity on the responsiveness of the different cell types to these treatments.

## 2. Materials and Methods

Chemicals were obtained from Sigma-Aldrich, Poole, UK unless otherwise stated.

### 2.1. Ethical Approval

Patients gave full consent and were anonymised by the Nottingham Health Sciences Biobank (study number ACP 94). This study was conducted according to the guidelines laid down in the Declaration of Helsinki and all procedures involving human patients were approved by the Nottingham University Hospital Research Ethics Committee (reference 04/Q2403/70). Written informed consent was obtained from all patients.

### 2.2. Liver Tissue Preparation and Primary Human Hepatocyte Culture

Human liver tissue was obtained from patients undergoing liver resection for secondary liver cancer. All liver sections used were histologically normal and showed no sign of steatosis. The fresh liver segment was cannulated and subjected to a collagenase-based perfusion method to isolate viable hepatocyte cells, based on an original method developed to isolate pig hepatocytes [20]. These were seeded into collagen coated 96-well plates in plating medium consisting of Williams Medium E with 5 mM glucose, 10% (*v*/*v*) foetal bovine serum (FBS) and hepatocyte supplements (5 mM nicotinamide, 1.3 mM zinc sulphate, 0.6 mM copper sulphate, 12 μM dexamethasone, 15 μM sodium selenite, 5 μg/mL transferrin, 10 nM human insulin, 1 mM L-carnitine, 50 μg/mL gentamicin, 0.1% bovine serum albumin (BSA) and sodium bicarbonate to pH 7.4). After 15–16 h, PHH were then incubated for 4 h in FBS-free medium (above media without the FBS) before adding treatment media (see below). In total cells from 6 different livers were used in the study and allocated to experiment 1 and 2 (described below) depending on number of cells available.

### 2.3. Hepatoma Cell Line Culture

HepG2 (human hepatoblastoma–derived cell line) and McA (rat hepatocellular carcinoma cell line) were obtained from American Tissue Culture Collection and Huh7 (human hepatoblastoma–derived cell line) from JCRB Cell Bank. Cells were maintained individually in T75 flasks in high glucose (25 mM) Dulbecco’s Modified Eagle Medium containing 10% FBS, 100 U/mL penicillin, 100 μg/mL streptomycin and 2 mM L-glutamine. Cells were passaged every 3–7 days. Incubators were set at 37 °C and 5% CO_2_.

### 2.4. Treatment of Cells and Measurement of Lipid Accumulation

Cells were seeded onto collagen coated 96-well plates at densities of 1.25 × 10^4^ (HepG2), 1.5 × 10^4^ (Huh7), 2 × 10^4^ (McA) or 4.5 × 10^4^ (PHH) cells per well. These densities were determined to ensure a confluent monolayer by the end of the experiment. Cells were plated in Williams Medium E with hepatocyte supplements (see above) for all cell types for consistency. After approximately 18 h in FBS containing medium, the cell lines were washed with PBS before adding FBS-free treatment media.

In experiment 1, PHH and hepatoma cell lines were treated with different combinations of fatty acids, glucose and fructose in the presence of 10 nM insulin. For PHH, cells from each liver were plated on 2 separate plates and each treatment was replicated in 5 wells/plate (total replication = 10 per liver). The experiment was performed on cells from 5 different livers (A, B, D, E and F as identified in Table 1). For other cell lines 3 separate experiments were performed and in each case cells were plated onto 2 separate plates and each treatment was replicated in 5 wells/plate (total replication = 30). Fatty acids (200 μM) were added as a combination of palmitic, oleic and linoleic acids bound to BSA [21] at a ratio of 2:2:1. Each experiment was performed in the presence of 5 or 11 mM glucose and 0, 2 or 8 mM fructose and 0 or 200 μM fatty acids. In experiment 2, PHH, HepG2 and McA cells were treated with different combinations of insulin, fatty acids and glucose, with fatty acids and glucose being the same concentrations as in the first experiments, while insulin was added at 0, 5 or 10 nM. The decision was made not to include Huh7 cells in this experiment as determination of insulin sensitivity (as determined by response of the ratio of pAKT to total AKT, as described below) was found to be similar to HepG2 cells. Replication was the same as in the first experiment except that PHH were prepared from 3 separate livers (A, C and D as identified in Table 1). In all experiments, after 24 h incubation the media were refreshed and Nile Red assays were performed after 48 h.

Nile Red is a fluorescent stain used to detect intracellular lipid droplets, with increased specificity for neutral lipid at yellow gold fluorescence than red fluorescence [22]. 1 mM Nile Red in DMSO, with 10 mg/mL Pluronic F-127, was diluted in Hank’s Balanced Salt Solution (HBSS) to 30 μM immediately before use and protected from light. Cells were washed twice with HBSS before adding 100 μL of Nile Red to each well. The plate was left to incubate in the dark for 15 min. After a further wash, the cells were covered with HBSS before measuring fluorescence on a microplate reader at yellow gold fluorescence wavelengths (excitation 485 nm, emission 590 nm).

To normalise for cell number, DNA was quantified using the Hoechst 33,258 dye method [21,23]. Following determination of Nile Red staining, the HBSS was removed from each well and water added instead, then the 96-well plates were subjected to freezing (−20 °C) and thawing to lyse the cells. A working dye solution was prepared by diluting 1 mg/mL Bisbenzimide to 2 μg/mL in 2 × Tris-HCl NaCl EDTA (TNE) buffer [21,23] and 100 μL of the diluted dye added to each well on the thawed plate and the plate read on a microplate reader (excitation 355 nm, emission 460 nm). The amount of DNA per well was quantified using a standard curve, produced using a serial dilution of calf thymus DNA.

Nile Red fluorescence was expressed as fluorescence per μg DNA, and expressed as fold change value relative to the control on the same plate (i.e., 10 nM Insulin, 5 mM glucose, 0 mM fructose and 0 μM fatty acid for the fructose experiments and 0 nM Insulin, 5 mM glucose, 0 mM fructose and 0 μM fatty acid for the insulin experiments).

### 2.5. Determination of Insulin Sensitivity of Hepatoma Cell Lines

As before, HepG2, Huh7 and McA cells were seeded into collagen coated 12-well plates in the same plating medium but all at a density of 3 × 10^5^ cells per well. After approximately 18 h, the insulin and FBS were removed for 5 h, then half the wells (n = 3 per cell line) were stimulated with 100 nM insulin for 15 min as previously described [15], and the other half had the insulin-free media refreshed (n = 3 per cell line). The cells were harvested for Western blotting analysis to determine AKT phosphorylation, as well as protein quantification using a 2D Quant assay (GE Healthcare, Chicago, IL, USA). Samples were split across three 4–15% Criterion TGX gels (BioRad, Hercules, CA, USA) and equal protein was loaded. The blots were first probed using the primary antibody, rabbit anti-phospho-AKT (Ser473, #9271), followed by the secondary antibody, anti-rabbit IgG HRP-linked antibody (#7074, Cell Signalling Technologies, Leiden, The Netherlands) which was detected by chemiluminescence (ECL Select, Amersham, Marlborough, MA, USA) and imaged on the ChemiDoc MP (BioRad). Membranes were then submerged in stripping buffer and rocked for 15 min, then washed for 5 × 5 min with Tris-buffered saline with 0.1% Tween, before being re-probed with the secondary antibody to check there was no anti-pAKT remaining. After stripping the blots, they were re-probed with rabbit anti-AKT (#9272, Cell Signalling Technologies) and the same anti-rabbit secondary antibody, before detection and re-imaging as before.

### 2.6. Statistical Analysis

Statistical analyses were carried out using Genstat 21st Edition. Data for PHH was analysed by three-way ANOVA (glucose × fructose × fatty acid or insulin × glucose × fatty acid) with blocking for plate and liver. Data for cell lines was analysed by three-way ANOVA with blocking for plate. Two-way ANOVA (cell type × insulin), with blocking for gel, was used for AKT phosphorylation experiments. The cell lines and primary cells could not be combined into one analysis due to the problem of technical versus biological replication and determination of fluorescence at different gains. Power calculations indicated that to detect a treatment difference of 0.3, at a significance level of 0.050, with a power of 0.800, using a two-sided test, required a replication of 22. Technical replication was n = 30 for each cell line and n = 50 for all PHH combined (experiment 1) and n = 30 for experiment 2. It was not possible to increase biological replication for PHH due to the availability of liver samples. Significance was attributed at *p* < 0.05. Data for the ratio of phosphor-AKT/AKT was analysed by 2-way ANOVA (cell type × insulin).

## 3. Results

**Figure 1:**
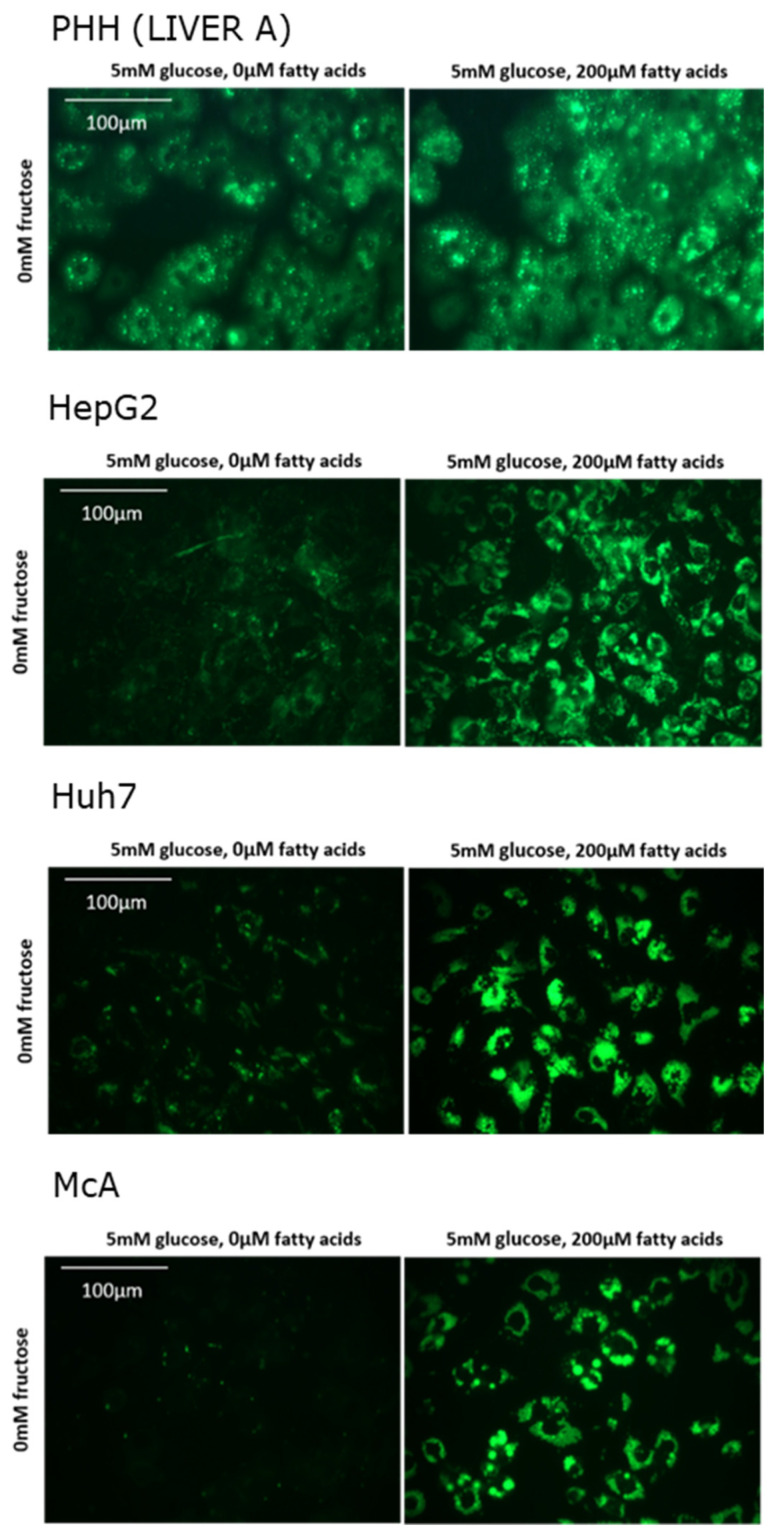
Effect of fatty acids on lipid accumulation in in different cell types. Lipid was visualised by staining with Nile Red and viewed under green fluorescence.

In total, PHH were isolated from 6 different individuals of both sexes and of varying age (Table 1). Histological examination of each of the samples indicated than none had overt steatosis. Figure 1 shows fluorescence images representative of each cell type in the absence and presence of fatty acids. It clearly shows that, in the absence of fatty acids, substantially more intracellular lipids were present in PHH than in hepatoma cell lines. Addition of fatty acids to the media increased the lipid content of all cell types.

Table 1 shows the mean fluorescence values for PHH from each of the patients studied and for each of the cell lines in the presence and absence of added fatty acids. Up to a three-fold variation could be seen for PHH isolated from different individuals when incubated in the absence of fatty acids. However, in all cases lipid content increased with the addition of fatty acids to the media. The size of this response varied between individuals with the greatest fold-changes seen in those with the lowest basal lipid levels. Overall, the average increase in lipid content in PHH was approximately two-fold. Fluorescence values for each cell line cannot be directly compared to PHH due to the requirement for different gain settings on the Fluorometer (due to very low basal values). Basal lipid levels were substantially lower in McA compared to both HepG2 and Huh7. All three cell lines accumulated significant amounts of lipid in response to fatty acid treatment with McA showing the greatest fold change.

**Figure 2:**
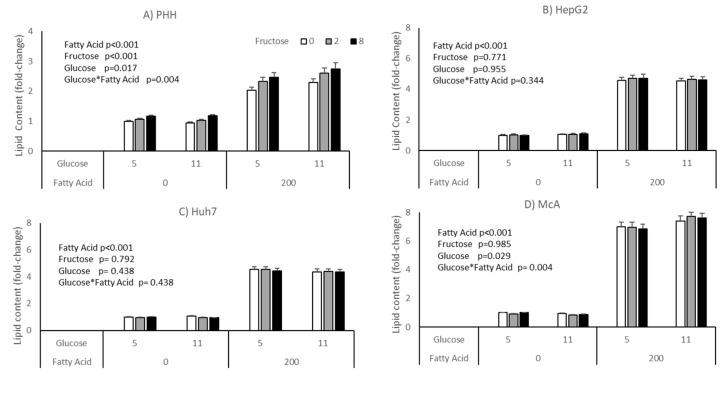
Impact of glucose, fructose and fatty acids on lipid accumulation in different cell types; (**A**) PHH, (**B**) HepG2, (**C**) Huh7, (**D**) McA. Values for PHH represent the mean (±SEM) of data from 5 individual livers (each experiment performed on 2 separate plates with 5 replicates per plate, total replicates = 50). Values for other cell lines (represent mean of data from 3 individual experiments (with similar replication as PHH, total replicates = 30/cell type). Data was analysed by 3-way ANOVA (glucose*fructose*fatty acid) with blocking for liver (for PHH) and plate for all cell types. No significant interactions were seen between Fructose*Glucose, Fatty Acids*Fructose or Fatty Acids*Fructose*Glucose.

The impact of glucose and fructose on lipid accumulation varied between cell types (Figure 2). Neither HepG2 nor Huh7 responded to either sugar, either in the presence or absence of fatty acids. Both McA and PHH showed a significant interaction between glucose and fatty acids (*p* = 0.004 for both), with lipid accumulation being higher in cells treated with fatty acids in the presence of 11 mM compared with 5 mM glucose. Only PHH showed a response to fructose, with intracellular lipid increasing with increasing concentrations independent of the concentration of glucose or addition of fatty acids. However, it should be noted that there were large variations in response of PHH from different individuals (Supplementary Table S1), with hepatocytes from only 2 of the 5 donors studied showing a statistically significant response to fructose. It should be noted that these were the two hepatocyte cultures with the lowest basal lipid content.

**Figure 3:**
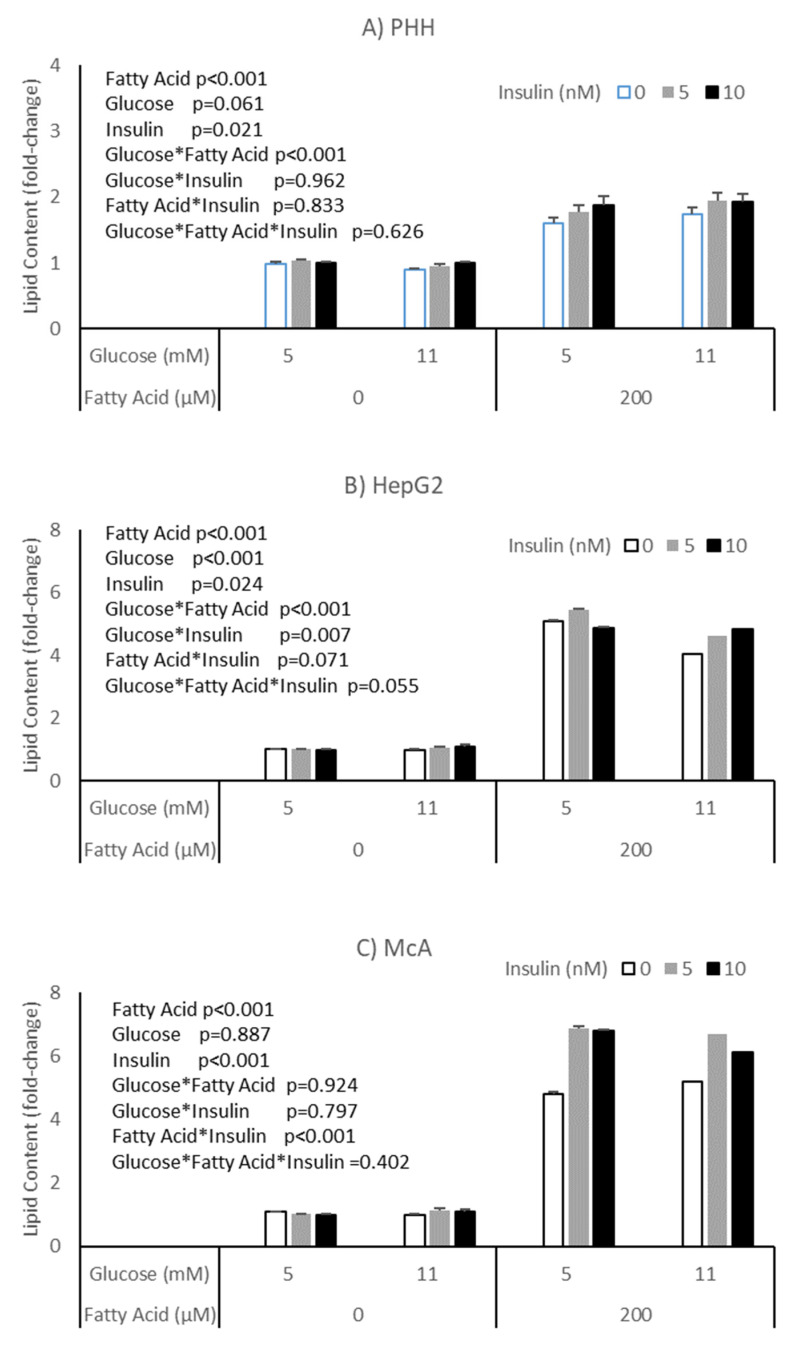
Impact of insulin on the response of different cell types to glucose and fatty acids; (**A**) PHH, (**B**) HepG2, (**C**) McA. Values for PHH represent the mean (±SEM) of data from 3 individual livers (each experiment performed on 2 separate plates with 5 replicates per plate, total replicates = 30). Values for other cell lines represent mean of data from 3 individual experiments (with similar replication as PHH, total replicates = 30/cell type). Data was analysed by 3-way ANOVA (insulin*glucose*fatty acid) with blocking for liver (for PHH) and plate for all cell types.

The potential impact of insulin on the effects of glucose and fatty acids on accumulation of lipid was explored in PHH, HepG2 and McA cells (Figure 3). All cell types responded to insulin with an increase in lipid, independent of glucose or fatty acid concentrations (*p* = 0.003, *p* = 0.024 and *p* < 0.001, respectively). McA showed the greatest response of the cell types studied and again there was variation between PHH from different livers, with two of the three studied showing a significant effect (Supplementary Table S2). Again, it was the hepatocyte culture with the highest basal lipid concentration (patient C) that failed to show an effect.

**Figure 4:**
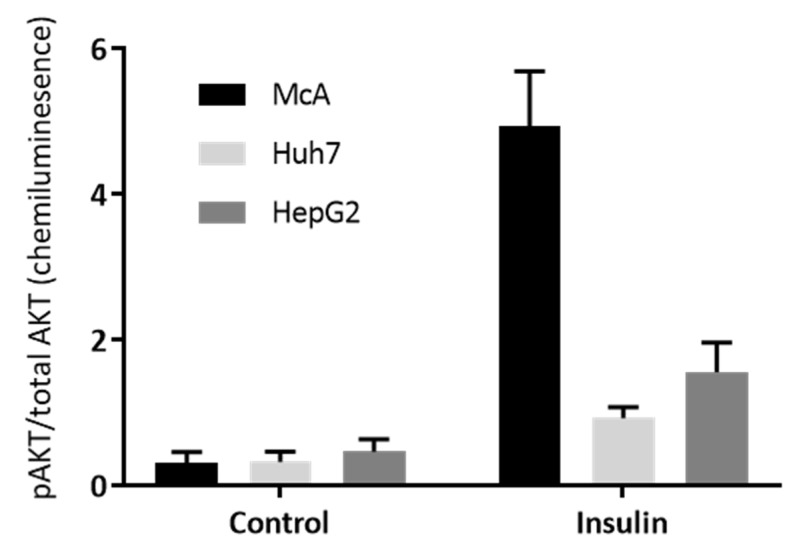
Effect of insulin on the ratio of pAKT to Total AKT in the three Hepatoma cell lines. Insulin sensitivity of McA, Huh7 and HepG2 cells determined by Western blotting for phosphorylated AKT (pAKT) and total AKT. Each cell type was probe on 3 separate gels/antibody (*n* = 1 per cell type on each gel) and data presented represents mean (±SEM) of the ratio of pAKT/total AKT (n = 3/cell type). Data was analysed by 2-way ANOVA (and a significant interaction between cell type and presence of insulin (*p* < 0.001) was found.

To further explore the insulin sensitivity of the three hepatoma cell lines, the phosphorylation of AKT in response to insulin was explored by Western Blotting. Supplementary Figure S1 shows the Western Blots and this data was used to calculate the ratio of phospho- (pAKT) to total AKT (Figure 4). There was a significant insulin × cell line interaction (*p* < 0.001) with the McA cells showing the biggest increase in the ratio with insulin treatment, indicating that McA cells were considerably more insulin sensitive than the other two cell lines.

## 4. Discussion

While it is well established that NAFLD is associated with insulin resistance there is also evidence that specific components of the diet can contribute to its development. Animal [6] and human [7,8,24] studies have suggested that diets rich in fructose may promote hepatic steatosis and its progression to steatohepatitis, to a greater extent than glucose. Multiple mechanisms have been suggested for this response including direct effects on hepatic lipogenesis [13]. For example, a recent study in healthy men showed that beverages sweetened with fructose or sucrose, but not glucose, promoted hepatic fatty acid synthesis [25]. Cell culture studies offer the opportunity to better understand the specific mechanisms whereby different substrates may impact on hepatic lipid metabolism. This includes both primary hepatocytes isolated from human or animal livers, as well as various hepatoma cell lines. While the potential value of such models has been reviewed [14,26] we believe this is the first study to directly compare PHH with three different hepatoma cell lines.

While it was not possible to quantitatively compare the amount of lipid in PHH and hepatoma cell lines (due to different gain settings), the captured images clearly show that the hepatoma cells accumulated much less lipid in control, fatty acid–free media compared to PHH. Furthermore, PHH from different individuals clearly showed marked differences in the amount of lipid accumulatedin the absence of added fatty acids. While histological examination of donor liver samples suggested that none of the samples used were clinically steatotic, both genetic and phenotypic factors could influence intracellular lipid content.

All four cell types accumulated lipid consistently and reproducibly after 48 h treatment with 200 μM fatty acids. This ability of fatty acids to induce lipid accumulation in hepatoma cell lines and primary hepatocytes is well characterised [15,16,17,27]. Lipid accumulation experiments using hepatoma cell lines have commonly used a combination of oleic and palmitic acids [15,17,28], but we believe the 40:40:20% palmitic:oleic:linoleic acid ratio used in the current study is more representative of the SFA:MUFA:PUFA ratio found in human blood [29]. Additionally, many in vitro studies are performed in the presence of FBS potentially containing varying amounts of insulin and other hormones as well as lipids. We therefore decided to use serum-free media supplemented with defined concentrations of insulin. Despite this, there were still differences in the amount of lipid accumulated in PHH in response to fatty acid treatment.

In contrast to the relatively consistent effects of fatty acids on the different cell types, the effects of glucose and fructose were very different. PHH and McA both showed fatty acid × glucose interactions, with an increase in lipid accumulation between 5 mM and 11 mM glucose only seen when fatty acids were present. However, it is important to note that this effect was inconsistent across PHH from the 5 livers, again suggesting genetic or phenotypic factors have the potential to impact on this response. By contrast, no effect of glucose was seen on lipid accumulation in HepG2 or Huh7 cells. Previous studies have shown HepG2 cells accumulate intracellular lipid with increased glucose treatment, but these have used supra-physiological glucose concentrations in excess of 20 mM. For example, Green et al. [30] and Hao et al. [31] both showed increased lipid accumulation in HepG2 cells with high (25 mM) versus low (5.5 mM) glucose. There are a number of possible reasons why different cell types (and PHH) may respond differently to glucose. HepG2 and Huh7 cells both express Hexokinase II rather than Glucokinase which is expressed in primary hepatocytes [32]. Recent studies indicate that switching from Hexokinase to Glucokinase in hepatoma cell lines elevates levels of lipogenesis and restores mitochondrial respiration and VLDL secretion [33]. Thus this difference in the enzyme responsible for the first step in glycolysis between hepatoma and primary cells, may in part explain the lack of lipogenesis in response to glucose. Fatty acids could provide a higher excess of energy substrate, so less carbohydrates might be used to produce ATP and therefore be stored as triacylglycerol (TAG) instead. Importantly, cell lines are cancer-like and still proliferating, with cell proliferation often associated with increased glycolysis to provide energy and substrate for biosynthesis of other macromolecules needed for cell replication [34]. In contrast, PHH are terminally differentiated, meaning they do not proliferate, and differentiated cells are more able to utilise oxidative metabolism for energy release, despite a lower energy demand [34]. Hence, there may be a greater excess of carbohydrate in PHH that is more readily stored as TAG. Alternatively, fatty acids may have limited storage as TAG unless there is sufficient glycerol-3-phosphate available, meaning that increased glucose (or fructose) could be used to synthesise glycerol-3-phosphate and thereby increase TAG storage when fatty acids are present. It is less clear why McA should behave differently to the human hepatoma cell lines, though as discussed later, this may relate to the insulin sensitivity of the cells.

The results of the current study showed that while fructose increased lipid accumulation in PHH (though again with variation in response between individuals) it had no effect in any of the three hepatoma cell lines. Other studies [35,36] have observed increased lipid accumulation with fructose treatment in HepG2 cells, but these were again performed with supra-physiological concentrations (up to 25 mM). Hoang et al. [36] found that both glucose and fructose increased lipid accumulation in HepG2 cells, but once again the concentrations used were supra-physiological. To our knowledge, no studies to date have shown any effects of fructose on lipid accumulation in McA cells. The lack of an effect of fructose in the three hepatoma cell lines could, again, be because of the cancerous phenotype of hepatoma cell lines. For example, aldolase B activity is very low and hexokinase 2 mRNA is much higher in HepG2 and Huh7 cells compared to PHH, although the activity of fructokinase has been shown to be similar [36]. Due to these differences in metabolic capacity, the ATP depletion caused by fructose in PHH has not been observed in HepG2 or Huh7 cells. The fact that the cell lines express aldolase A rather than aldolase B and have higher expression of hexokinase 2 gives them more of a muscle phenotype in terms of fructose metabolism [37]. This could be one factor affecting the incorporation of fructose into lipid in these cells, with fructose being more readily channeled into glycolysis.

Another possible reason for the difference in response of cell types to glucose may be their sensitivity to insulin. To test this, we compared the response of PHH, HepG2 and McA cells to combinations of glucose and fatty acids in the presence of varying concentrations of insulin. All cell types showed an increase in lipid accumulation in response to insulin (independent of the presence of fatty acids or glucose concentration). Again, differences were seen in PHH from different individuals with only two of the three preparations appearing insulin sensitive. The response in McA cells was considerably higher than HepG2, suggesting that the differences in response in these two cell lines may be due to insulin sensitivity. This difference was subsequently confirmed when we compared AKT phosphorylation across the three hepatoma cell types, with McA cells appearing much more insulin sensitive than the other two cell types.

## 5. Conclusions

The main findings of this study are that the 3 hepatoma cell lines (HepG2, Huh7 and McA) all showed increased lipid accumulation with fatty acid treatment, but there were no effects of fructose in any cell line and only McA cells showed an increased lipid accumulation with glucose treatment (and only in the presence of fatty acids). In contrast, there were significant effects of fatty acids, glucose and fructose (including interactions) in the PHH, but the responses varied across the 5 livers used. Due to the low numbers of livers, it was not possible to identify what factors might be responsible for this observed variability between livers. However, both HepG2 and Huh7 cells showed lower insulin sensitivity (measured as Akt phosphorylation) compared to McA cells, which could be involved in the differences observed. Importantly, the results from this study suggest that the hepatoma cell lines are not a good model for PHH and therefore their continued use is questionable (particularly HepG2 and Huh7 cells). Future work needs to integrate nutritional and genetic factors in physiologically relevant models.

## Figures and Tables

**Table 1:** Comparison of lipid content of different cell types in absence and presence of added fatty acids.

Cells				Lipid Content (Fluorescence)					
			Gain *	0 FFA		200 FFA		Fold-Change	
Primary Hepatocytes				Mean	SE	Mean	SE	Mean	SE
	Sex	Age							
A	F	67	1500	47,626	5779	132,012	9301	2.771	0.117
B	M	63	1500	53,679	1613	125,418	3610	2.340	0.060
C	M	60	1500	132,933	7202	211,192	14,622	1.576	0.068
D	M	71	1500	151,565	10,819	171,891	6029	1.145	0.042
E	F	72	1500	71,190	2775	116,925	9018	1.647	0.132
F	F	25	1500	67,989	4682	154,560	10,340	2.275	0.131
Average				87,497	5764	152,000	5595	1.959	0.054
HepG2			1250	48,083	9127	219,481	10,886	4.587	0.235
Huh7			1250	36,405	1046	164,782	9631	4.534	0.279
McA			1250	11,442	350	93,332	7036	8.136	0.580

* Gain setting on FLUOstar Omega Plate Reader.

## Data Availability

Detailed data relating to the study is presented in Supplementary Tables S1 and S2.

## Supplementary Materials

The following supporting information can be downloaded at: https://www.mdpi.com/article/10.3390/nu15010040/s1, Table S1: Impact of glucose, fructose and fatty acids on lipid accumulation in primary human hepatocytes; Table S2: Impact of insulin on response of primary human hepatocytes to glucose and fatty acids. Figure S1: Insulin sensitivity of McA-RH7777, Huh7 and HepG2 cells determined by Western blotting for phosphorylated AKT (pAKT) and total AKT. Images of the three gels (n = 1 per cell type on each gel) probed with pAKT or total AKT antibodies, control (-) or insulin treated (+). - and + HepG2 cell protein was run on each gel in the last two lanes to demonstrate consistency.
