# Supplementary material for: A Comparison of Primary Human Hepatocytes and Hepatoma Cell Lines to Model the Effects of Fatty Acids, Fructose and Glucose on Liver Cell Lipid Accumulation
Source: Nutrients. 2022 Dec 21;15(1):40. doi: 10.3390/nu15010040 (PMC9824391; doi:10.3390/nu15010040)
Supplement: Supplementary file 1 [file nutrients-15-00040-s001.zip › nutrients-2095142-supplementary.pdf]

Supplementary Table S1: Impact of glucose, fructose and fatty acids on lipid accumulation in primary human hepatocytes

Liver			A		B		D		E		F		Average	
			Fluorescence											
Fatty Acid	Glucose	Fructose	mean	SE	mean	SE	mean	SE	mean	SE	mean	SE	mean	SE
0	5	0	47626	5779	53679	1613	151565	11324	71190	2775	67989	4682	78410	5999
0	5	2	46305	5186	67944	2193	149352	10235	76718	3783	72232	3738	82510	5574
0	5	8	49531	4619	82270	4269	140880	7995	79303	5666	81825	7102	86762	4985
0	11	0	42116	3856	51087	3009	120337	6576	75016	4202	69834	5030	71678	4366
0	11	2	46205	3402	62382	3543	125519	6838	74901	7080	78605	5310	77522	4456
0	11	8	59082	3060	77196	4951	134360	9979	77531	3285	84397	4154	86513	4365
200	5	0	132012	9301	125418	3610	171891	6029	116925	9018	154560	10340	140161	4497
200	5	2	149430	15049	163165	6638	184848	6990	125961	10026	167830	15040	158247	5627
200	5	8	190677	11145	165338	11881	179353	8657	117688	9389	160722	10743	162756	5711
200	11	0	150854	12180	124982	6882	195254	15555	135132	8051	182308	14247	157706	6386
200	11	2	203728	21475	145476	8265	213434	18556	136384	5986	181758	11329	176156	7571
200	11	8	226630	25746	176662	8150	209315	19485	135544	6188	156279	9720	180886	8269
3-way ANOVA (p-values)														
Fatty acid			<0.001		<0.001		<0.001		<0.001		<.0001		<0.001	
Fructose			<0.001		<0.001		0.397		0.623		0.54		<0.001	
Glucose			0.007		0.205		0.652		0.025		0.099		0.007	
Fatty acid*Fructose			0.003		0.009		0.531		0.686		0.066		0.075	
Fatty acid*Glucose			0.012		0.683		<0.001		0.026		0.361		<0.001	
Fructose*Glucose			0.441		0.067		0.487		0.725		0.393		0.847	
Fatty acid*Fructose*Glucose			0.607		0.089		0.785		0.902		0.378		0.887	

Values for individual livers represent means of 10 replicates (5 replicates on 2 plates). For each liver 3-way ANOVA was performed with blocking for plate for individual livers and plate*liver for averaged data.

Supplementary Table S2: Impact of insulin on response of primary human hepatocytes to glucose and fatty acids

Liver			A		C		D		Average	
			Fluorescence							
Fatty Acid	Glucose	Insulin	mean	SE	mean	SE	mean	SE	mean	SE
0	5	0	52631	2972	128000	6667	83235	2797	87955	6273
0	5	5	53353	1946	135493	6171	91794	2977	93547	6639
0	5	10	52972	2668	132933	7202	87495	3690	91133	6670
0	11	0	55720	2251	115118	6025	67599	3572	79479	5321
0	11	5	57172	3146	122132	6945	76221	4867	85175	5840
0	11	10	55720	2001	135077	7095	79928	5363	90390	6821
200	5	0	107916	6834	213602	17292	104777	5553	142098	11277
200	5	5	133167	6920	211576	12168	109088	3875	150931	9296
200	5	10	151402	8456	211192	14622	105781	5260	156125	9830
200	11	0	108126	3323	257688	17500	106965	6354	157593	14475
200	11	5	102943	1887	286239	19376	122787	7246	175515	16085
200	11	10	118079	6641	271975	17026	126407	6276	172153	14520

3-way ANOVA (p-values	A	C	D	Average
Fatty acid	<0.001	<.001	<.001	<.001
Glucose	0.024	<.001	0.885	0.061
Insulin	<0.001	0.446	<.001	0.021
Fatty acid*Glucose	<0.001	<.001	<.001	<.001
Fatty acid*Insulin	<0.001	0.789	0.937	0.586
Glucose*Insulin	0.055	0.583	0.035	0.833
Fatty acid*Glucose.Insulin	0.063	0.586	0.483	0.626

Values for individual livers represent means of 10 replicates (5 replicates on 2 plates). 3-way ANOVA was performed with blocking for plate for individual livers and plate*liver for averaged data

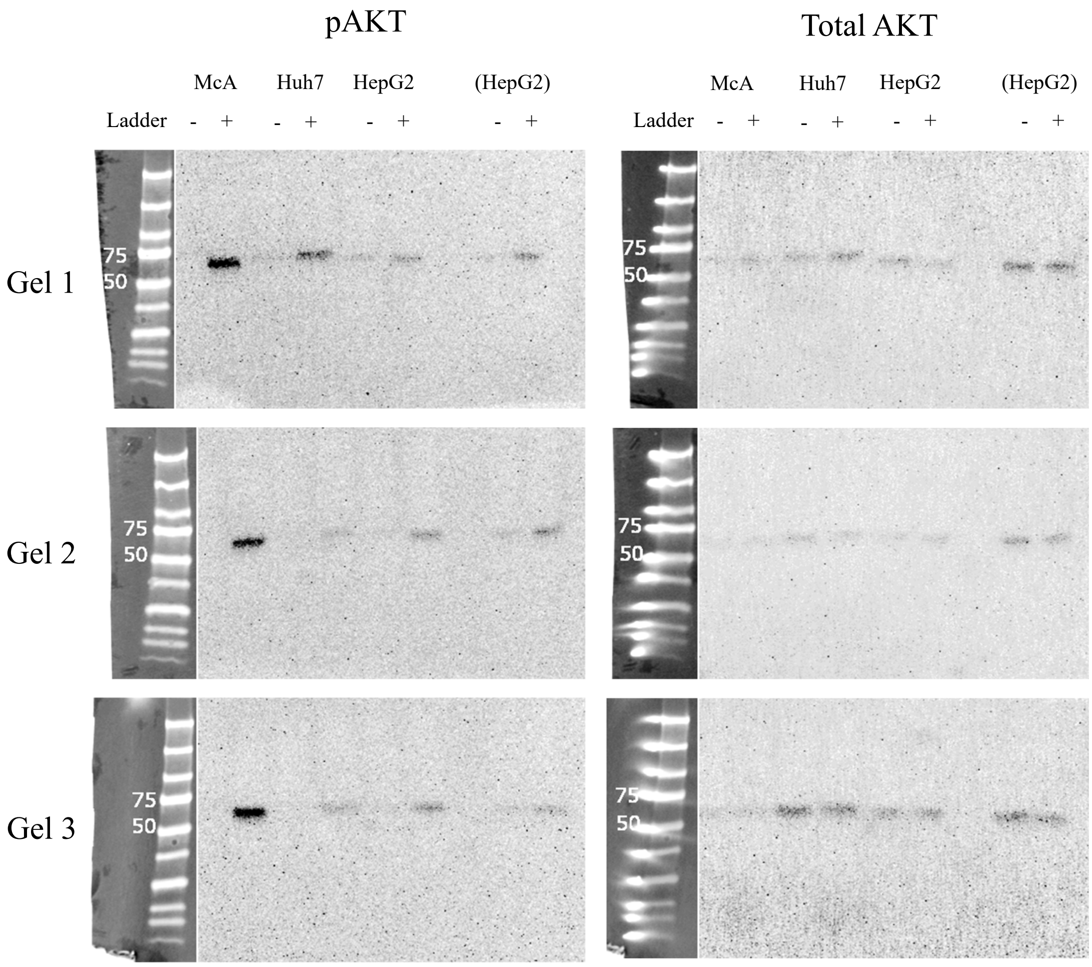

Supplementary Figure S1. Insulin sensitivity of McA-RH7777, Huh7 and HepG2 cells determined by Western blotting for phosphorylated AKT (pAKT) and total AKT. Images of the three gels (*n*=1 per cell type on each gel) probed with pAKT or total AKT antibodies, control (-) or insulin treated (+). - and + HepG2 cell protein was run on each gel in the last two lanes to demonstrate consistency.
